# Epidemiological characterization of respiratory tract infections caused by *Mycoplasma pneumoniae* during epidemic and post-epidemic periods in North China, from 2011 to 2016

**DOI:** 10.1186/s12879-018-3250-2

**Published:** 2018-07-17

**Authors:** Jiuxin Qu, Chunxia Yang, Fang Bao, Shuyan Chen, Li Gu, Bin Cao

**Affiliations:** 1 Department of Clinical Laboratory, The Third People’s Hospital of Shenzhen, Shenzhen, Guangdong China; 2 Department of Infectious Diseases and Clinical Microbiology, Beijing Chao-Yang Hospital, Beijing Institute of Respiratory Medicine, Capital Medical University, Beijing, China; 3 Department of Pediatrics, Beijing Chao-Yang Hospital, Beijing Institute of Respiratory Medicine, Capital Medical University, Beijing, China; 4 Department of Clinical Trial, The Third People’s Hospital of Shenzhen, Shenzhen, Guangdong China; 5 Department of Respiratory and Critical Care Medicine, Clinical Microbiology and Infectious Disease Lab, China-Japan Friendship Hospital, Beijing, China

**Keywords:** *Mycoplasma pneumoniae*, Respiratory tract infection, Epidemic, Post-epidemic, Epidemiology

## Abstract

**Background:**

*Mycoplasma pneumoniae* (*M. pneumoniae*) is a commonly causative pathogen for respiratory tract infections (RTIs) in humans. The epidemiological features of *M. pneumoniae* infections during post-epidemic, including age distribution and the seasonality of the patients, are not well investigated.

**Methods:**

We retrospectively analyzed the clinical data of 7835 consecutive RTIs patients (3852 adults and 3983 children) who visited a teaching hospital, and defined an epidemic (2011–2013) and a post-epidemic period (2014–2016). *M. pneumoniae* was detected by fluorescence-quantatitive PCR in respiratory samples. Informed consent was obtained by all adults and the legal representatives of patients aged < 18 years, and the study was approved by Institutional Review Board of Beijing Chao-Yang Hospital (project approval number 10-KE-49).

**Results:**

The median (IQR) age was 16 (53) years (range < 0–105 years). The *M. pneumoniae* positive rate was 14.4% (21.2%, epidemic; 6.7%, post-epidemic), with seasonal peaks from late summer to autumn during epidemic, and from fall to winter during post-epidemic period, which was highest in children aged 7–17 years. In epidemic, no statistical difference was found in the positive rates between children and adults among most months (except February, July and August), neither for the positive rates among age groups (*P* = 0.801). However, in post-epidemic period, significant differences were observed in the positive rates between children and adults in nearly every month (*P*< 0.05 or *P*< 0.001, except May), as well as in the positive rates among age groups (*P*< 0.001). Most of the older patient admissions and all of ICU admissions occurred during the epidemic.

**Conclusions:**

Different patterns of age distribution and seasonality of *M. pneumoniae* RTIs between epidemic and post-epidemic periods were reported. Our results suggest that *M. pneumoniae* should be considered as a possible pathogen in pneumonia patients admitted to the ICU in the setting of an epidemic.

**Electronic supplementary material:**

The online version of this article (10.1186/s12879-018-3250-2) contains supplementary material, which is available to authorized users.

## Background

*Mycoplasma pneumoniae* (*M. pneumoniae*) is a common pathogen of respiratory tract infections (RTIs) in humans, especially in children and young adults [1, 2]. *M. pneumoniae* infections rangle clinically from mild, self-limiting upper respiratory symptoms to radiographically confirmed pneumonia requiring hospitalization and account for 10–30% of community-acquired pneumonia (CAP) cases [2–5]. Real-time polymerase chain reaction (PCR) has emerged as the primary techniques for detection of *M. pneumoniae* in surveillance programs and clinical practices in China [6, 7], due to its increased sensitivity and specificity compared with culture-based and serological methods.

*M. pneumoniae* is the smallest self-living and cell wall-less bacterium. Droplet infection during close contact mediates transfer of this pathogen from person to person and can lead to an epidemic. Epidemics of *M. pneumoniae* infections are documented worldwide with durations of 3–5 years [8–10]. The fluctuations in the incidence of *M. pneumoniae* infections are considered due to a decline in a population’s immunity and an increase of the immunologically naïve population [11], or changes in the proportion of individual strains with specific genotypes. Many investigations have focused on the epidemics, individual cases, and small clusters and outbreaks with various sizes [10, 12–14]; however, epidemiological features of *M. pneumoniae* infections during post-epidemic or non-epidemic periods, including age distribution, the seasonality and the hospitalization rate of the patients, have not been studied in detail.

In this study, we retrospectively analyzed patients of all age with *M. pneumoniae* RTIs from 2011 to 2016, covering an epidemic (2011–2013) and a post-epidemic period (2014–2016), and explored the epidemiological patterns of the infections during the two periods.

## Methods

### Study population and samples

During January 2011 to December 2016, 8213 consecutive patients with RTIs who visited Beijing Chao-Yang Hospital were prescribed by physicians with a PCR detection on *M. pneumoniae*. For the 378 patients who were re-diagnosed with the infection within two months of the initial diagnosis, only the data from the first incidence was analyzed in this study. We retrospectively analyzed the data of 7835 patients, including age, gender, date of visit, sample type and site of care. The *M. pneumoniae* RTIs included upper tract infection, bronchitis, bronchopneumonia, and pneumonia. The sample types included oropharyngeal swab, sputum, bronchoalveolar lavage fluid (Balf) and others (pleural effusion, cerebrospinal fluid and tissue).

Informed consent was obtained from all adults and the legal representatives of patients aged < 18 years at admission for their clinical records to be used in future study. The study was approved by Institutional Review Board of Beijing Chao-Yang Hospital (project approval number 10-KE-49).

### Fluorescence quantitative PCR (FQ-PCR) testing of *M. pneumoniae*

DNA was extracted from 200 μl of clinical samples by manual nucleic acid extraction (Qiagen QIAmp DNA Mini Kit, Valencia, CA). As described by Qu et al. [7], *M. pneumoniae* was detected by a commercial FQ-PCR kit (Daan Gene, Guangzhou, China) approved by State Food and Drug Administration, targeting the 16S ribosomal RNA gene [GenBank: AF132740]. The FQ-PCR mixture was prepared in a total volume of 45 μl, containing 3 μl of sample DNA. FQ-PCR was performed under the following conditions: initial activation at 93 °C for 2 min, followed by 10 cycles at 93 °C for 45 s and 55 °C for 1 min, and 30 cycles at 93 °C for 30 s and 45 °C for 30 s. The qualitative data from this procedure was used for the diagnosis. An internal control targeting human ribonuclease protein (hRNP) gene was incorporated. The amplifications were performed using the AB 7500 Real Time PCR System (Applied Biosystems, Foster City, CA) according to the manufacturer’s instructions.

### Statistical analyses

Categorical variables were described with counts and percentages. Age was presented as median (IQR). The Wilcoxon rank sum test was used in comparison of age between groups and the χ^2^ or Fisher’s exact test was used in comparisons of categorical variables. Statistical analyses were performed with SPSS Statistics (v21, IBM Corp., USA). *P* value < 0.05 was considered significant.

## Results

### Demographic characteristics of the patients

Overall, the median (IQR) age was 16 (53) years (range< 0–105years), and the children (aged < 18years) to adults (aged ≥18 years) ratio was nearly 1:1 (3983:3852; Table 1). Oropharyngeal swab and sputum were the main samples, accounting for 66.3 and 29.2%, respectively. The peak infection rates and number of *M. pneumoniae* positive cases were both noted at the age of 2 to 8 years (Additional file 1: Figure S1); Most of the patients who had *M. pneumoniae* infections (14.4%, 1127/7835) were children (19.7% vs. 8.9%, *P*< 0.001) and female (15.7% vs. 13.3%, *P* = 0.003). Of the infected cases, 531/1127 (47.1%) and 582/1127 (51.6%) patients with mild and mediate severity were treated as outpatients or in wards, only 14/1127 (1.2%) patients were treated in Respiratory ICU (RICU) and Emergency ICU (EICU) or other ICUs.

**Table 1:** Characteristic analysis on *M. pneumoniae* cases during 2011–2016. Data were represented as n (%) in total cases, and as n (positivity, %) in *M. pneumoniae* cases

Characteristic	Total	*M. pneumoniae* cases	*P* value
Age, median (IQR)	16 (53)	11 (19)	
Children	3983 (50.8)	786 (19.7)	< 0.001
Adults	3852 (49.2)	341 (8.9)	
Gender			0.003
Female	3514 (44.9)	552 (15.7)	
Male	4321 (55.1)	575 (13.3)	
Sample types			< 0.001
Oropharyngeal swab	5196 (66.3)	883 (17.0)	
Sputum	2290 (29.2)	225 (9.8)	
Balf ^a^	332 (4.2)	19 (5.7)	
Others	17 (0.3)	0 (0)	
Site of care			< 0.001
Outpatients	3427 (43.7)	531 (15.5)	
Wards	3771 (48.1)	582 (15.4)	
RICU and EICU	357 (4.6)	11 (3.1)	
Other ICUs	280 (3.6)	3 (1.1)	
Total	7835	1127 (14.4)	

^a^Balf: bronchoalveolar lavage fluid

*P* value: statistical analyses were performed within *M. pneumoniae* cases

### Annual cases from 2011 to 2016

Annual cases and the positive rate of *M. pneumoniae* during the study period are shown in Additional file 2: Figure. S2. The positive rate of *M. pneumoniae* during 2011 to 2013 was much higher than that during 2014 to 2016 (21.2% vs. 6.7%), on the basis of equal numbers of detected patients (3998 vs. 3837). To exclude the influence of seasonality on monthly distribution of *M. pneumoniae* cases, in this study, we defined an epidemic (during 2011 to 2013) and a post-epidemic period (during 2014 to 2016) by year.

### Monthly analyses on *M. pneumoniae* cases

**Fig. 1:**
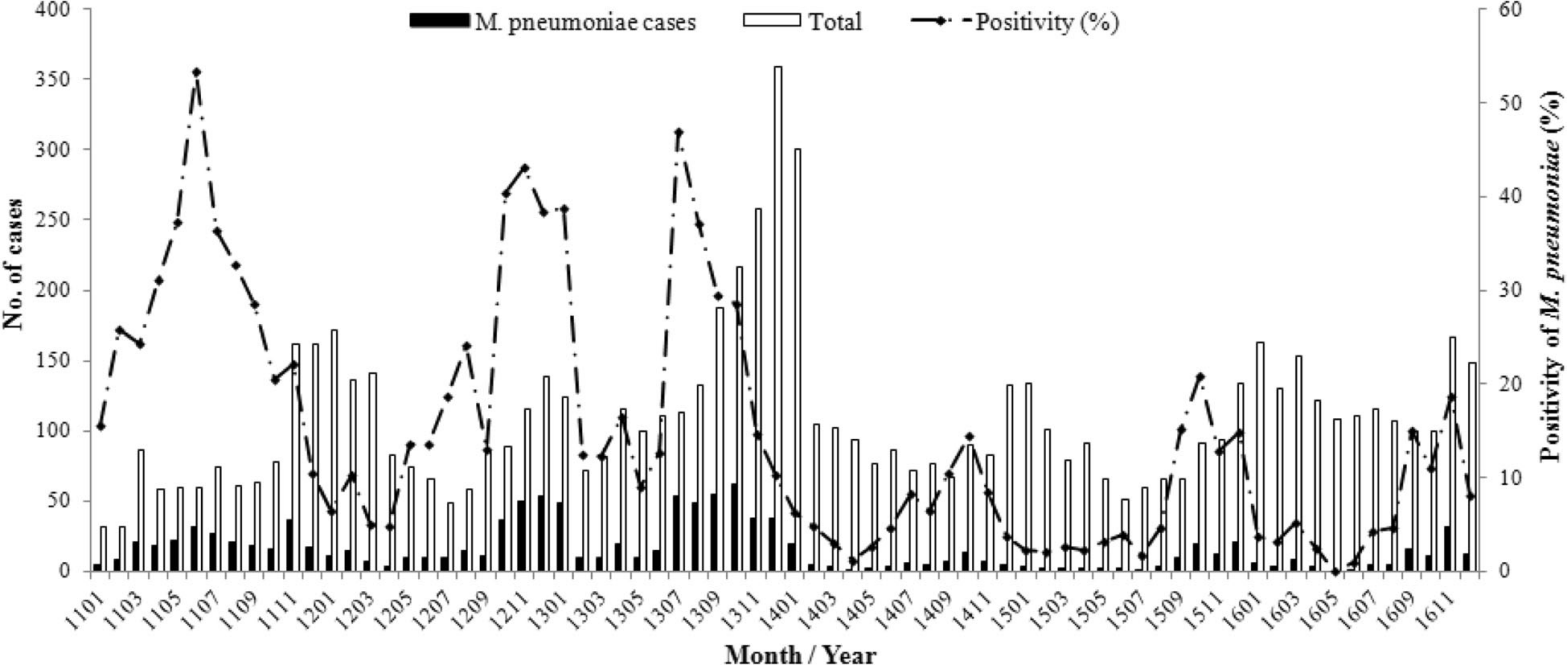
Monthly distribution of total cases and *M. pneumoniae* infected patients

**Fig. 2:**
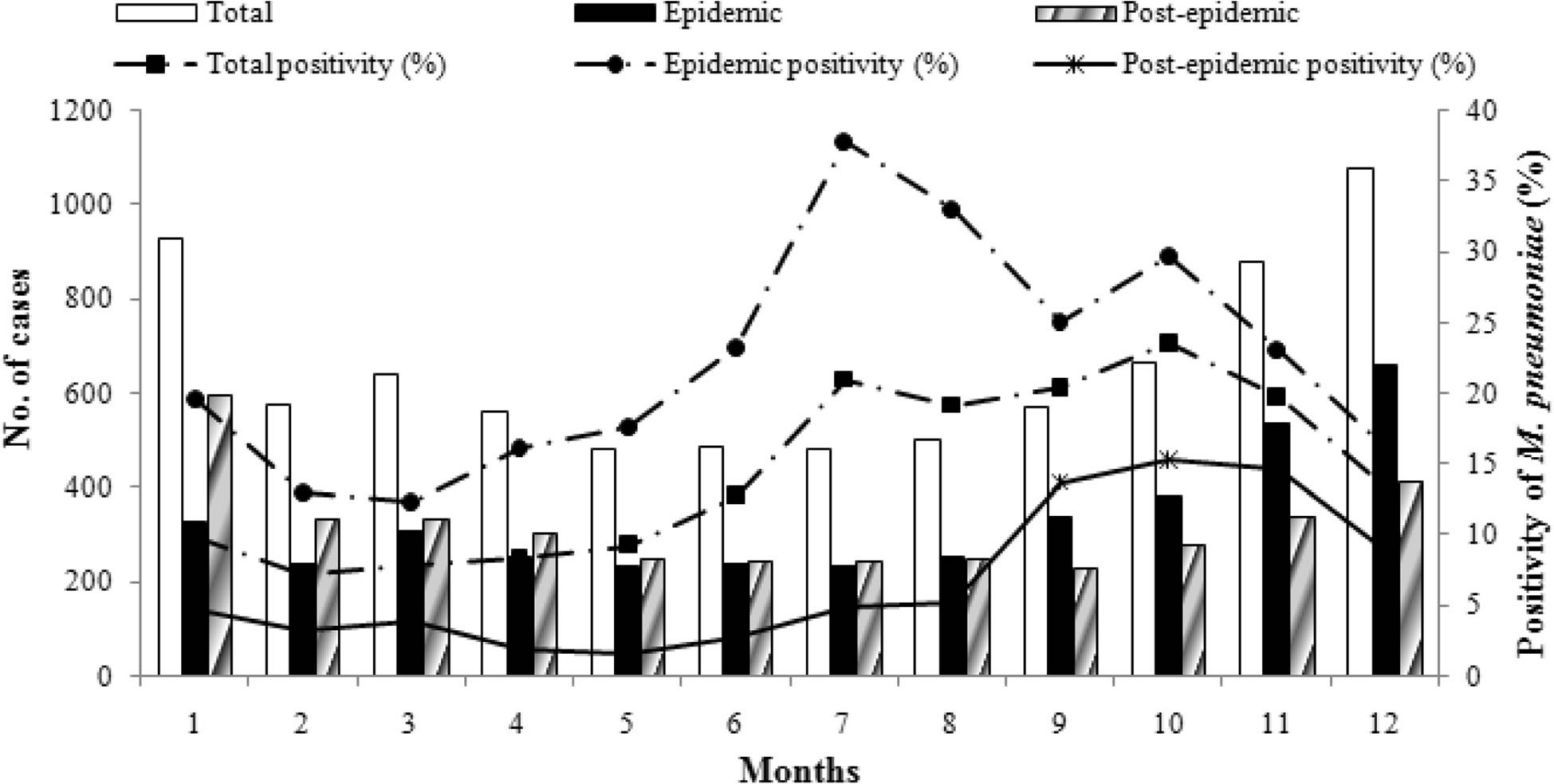
Monthly cases and positivity of *M. pneumoniae* in the epidemic, post-epidemic and the study periods

The monthly cases for the total study period are shown in Fig. 1. During the epidemic, there were three peaks with the positive rate of *M. pneumoniae* over 30%, increased from late summer to autumn and lasted for 4 to 5 months. The lowest positive rate of *M. pneumoniae* was seen as 5% in March 2012. During the post-epidemic period, the largest number of *M. pneumoniae* cases was observed in autumn and winter, which coincided with the positive rate of *M. pneumoniae*. The positive rate of *M. pneumoniae* was lower than 2% in several single months, even no detections in May 2016. When both of the epidemic and post-epidemic merged into 12 months from January to December, the *M. pneumoniae* positivity values for every month during the epidemic was higher than those during the post-epidemic period (Fig. 2).

As shown in Additional file 3: Table S1, the *M. pneumoniae* positivity of children was significantly higher than that of adults almost in every month, except May and June. Both of the peaks of positive rate in children and adults were from July to November (Additional file 4: Figure. S3). When we analyzed the data divided by epidemic and post-epidemic, we found interesting epidemiological patterns of monthly *M. pneumoniae* positivity between children and adults (Additional file 5: Table S2 and Additional file 6: Figure S4). In epidemic, the *M. pneumoniae* positivity of children was significantly higher than that of adults only in February, July and August (*P*=0.025, *P*< 0.001 and *P*< 0.001). The differences in *M. pneumoniae* positivity were not statistically significant in most months, the positive rates of adults were even higher than that of children in June and December. Whereas during post-epidemic period, the *M. pneumoniae* positivity of children was significantly higher than that of adults in nearly every month, except May.

### Analyses on *M. pneumoniae* cases by age groups

The patients were also divided into eight groups by age: 0–1 year (infants), 2–3 years (toddlers), 4–6 years (pre-school children), 7–17 years (school children), 18–44 years (young adults), 45–64 years (older adults), 65–79 years (the elderly) and ≥ 80 years (very elderly). As shown in Table 2 and Additional file 7: Figure S5, during the study period, the *M. pneumoniae* positivity increased from 8.2% of infants to the highest 32.9% of school children, then dropped steeply from 18.8% of young adults to 2.5 and 1.7% of elderly patients (*P*< 0.001). Similar trends of *M. pneumoniae* positivity were observed in epidemic and post-epidemic periods. However, the *M. pneumoniae* positivity among the age groups did not differ significantly (*P* = 0.801) in epidemic. Most of *M. pneumoniae* infected patients aged over 45 years occurred during epidemic.

**Table 2:** Analysis of *M. pneumoniae* positivity among patients during epidemic and post-epidemic by age groups. Data were represented as n (%) in total cases, and as n (positivity, %) in *M. pneumoniae* cases

Age group	Total	*M. pneumoniae* cases	*P* value	Epidemic		*P* value	Post-epidemic		*P* value
				Total	*M. pneumoniae* cases		Total	*M. pneumoniae* cases	
Infants	415	34 (8.2)	< 0.001	246	30 (12.2)	0.801	169	4 (2.4)	< 0.001
Toddlers	1172	127 (10.8)		768	113 (14.7)		404	14 (3.5)	
Pre-school children	1048	182 (17.4)		671	136 (20.3)		377	46 (12.2)	
School children	1348	443 (32.9)		849	325 (38.3)		499	118 (23.6)	
Young adults	1260	237 (18.8)		677	182 (26.9)		583	55 (9.4)	
Older adults	1254	74 (5.9)		432	59 (13.7)		822	15 (1.8)	
The elderly	928	23 (2.5)		271	19 (7.0)		657	4 (0.6)	
Very elderly	410	7 (1.7)		84	7 (8.3)		326	0 (0)	
Total	7835	1127 (14.4)		3998	871 (21.8)		3837	256 (6.7)	

*P* value: statistical analyses were performed on *M. pneumoniae* cases among age groups

### Analyses on positive sample type and site of care during epidemic and post-epidemic

As shown in Table 3, the *M. pneumoniae* positivity of the main samples (swab, sputum and balf) was nearly equal (all over 20%, *P* = 0.39) during the epidemic, with the highest rate in balf (27.8%). The *M. pneumoniae* positivity in post-epidemic was significantly different among the samples (*P*< 0.001), which decreased from upper (9.2%) to lower tract samples (4.6 and 1.4%). When analyzed the patients by site of care, the *M. pneumoniae* positivity between outpatients and inpatients was almost equal no matter in epidemic (21.1% vs. 23.6%) or in post-epidemic (7.2% vs. 7.9%). All *M. pneumoniae* cases that required treatment in ICUs occurred only during the epidemic.

**Table 3:** Characteristic analysis on *M. pneumoniae* cases during epidemic and post-epidemic. Data were represented as n (%) in total cases, and as n (positivity, %) in *M. pneumoniae* cases

Characteristic	Epidemic			Post-epidemic		
	Total	*M. pneumoniae* cases	*P* value	Total	*M. pneumoniae* cases	*P* value
Age, median (IQR)	9 (30)	11 (20)		39 (58)	10 (19)	
Children	2534 (63.4)	604 (23.8)	< 0.001	1449 (37.8)	182 (12.6)	< 0.001
Adults	1464 (36.6)	267 (18.2)		2388 (62.2)	74 (3.1)	
Gender			0.145			0.102
Female	1889 (47.2)	431 (22.8)		1625 (42.4)	121 (7.4)	
Male	2109 (52.8)	440 (20.9)		2212 (57.6)	135 (6.1)	
Sample types			0.39			< 0.001
Oropharyngeal swab	3265 (81.7)	705 (21.6)		1931 (50.3)	178 (9.2)	
Sputum	675 (16.9)	151 (22.4)		1615 (42.1)	74 (4.6)	
Balf ^a^	54 (1.4)	15 (27.8)		278 (7.2)	4 (1.4)	
Others	4 (0.1)	0 (0)		13 (0.4)	0 (0)	
Site of care			0.581			< 0.001
Outpatients	2041 (51.1)	431 (21.1)		1386 (36.1)	100 (7.2)	
Wards	1807 (45.2)	426 (23.6)		1964 (51.2)	156 (7.9)	
RICU and EICU	96 (2.4)	11 (11.5)		261 (6.8)	0 (0)	
Other ICUs	54 (1.4)	3 (5.6)		226 (5.9)	0 (0)	

^a^Balf: bronchoalveolar lavage fluid

*P* value: statistical analyses were performed within *M. pneumoniae* cases

## Discussion

From 2011 to 2016, we observed that 14.4% (1127/7835) of patients with RTIs were positive for *M. pneumoniae*, which was comparable with the global incidence as 12% (range 11–15%) from the Atypical Pathogens Reference Laboratory Database [15]. Similar to previous reports [16, 17], the positive rates rose during the late summer and autumn and returned to low values during late winter and spring. The *M. pneumoniae* positive rates of children was significantly higher than those of adults (19.7% vs. 8.9%, *P*< 0.001) nearly in every month (Additional file 3: Table S1), and the highest rate was found in the school children group aged 7–17 years (32.9%, 443/1348). Similarly, Kogoj et al. reported the proportion of *M. pneumoniae* RTIs were 19 and 7% in children and adults, respectively, and the highest proportion was found in patients aged 6–16 years (26%; 565/2162) [18]. These observations were consistent with the fact that the majority of outbreaks had occurred within a community or in closed or semi-closed settings such as military bases or schools. Moreover, compared with other publications on hospitalized patients [17, 19] and pediatric patients [17, 20–22], these results were more comprehensive to the patients with different severity and age because we reported on patients with 43.7% (3427/7835) as outpatients and adults to children ratio as 1:1 (3852:3983).

We firstly characterized the epidemiological features of *M. pneumoniae* infections during the epidemic and post-epidemic periods. Slightly different seasonality was displayed between the two periods: the positive rates peaked from late summer to autumn in epidemic period, and from fall to winter in the post-epidemic period. A positive correlation between increases in temperature and the occurrence of *M. pneumoniae* infections was reported by Onozuka and colleagues [23, 24], which might help explain the higher numbers that occur during warmer months.

The positive rate trends among age groups were similar between the two periods, with higher rates in patients aged 4–44 years old. The distribution corresponds to the findings from England and Wales [4, 25]. However, *M. pneumoniae* positive rate was significantly higher in pre-school and school children during post-epidemic period (*P*< 0.001; Table 2), and no statistical difference was found during epidemic period (*P*=0.801; Table 2). Furthermore, most of older and elderly cases occurred during the epidemic period. A single genotype of *M. pneumoniae* is not the probable cause of the epidemic, since other studies have shown that both endemic and epidemic spreads of *M. pneumoniae* may be polyclonal [11, 26, 27]. Therefore, the potential reason for the observed pattern of age distribution during the two periods may be related to interactions between the pathogen and the immunological status of the human population [28]. A mathematical model of this process was recently reported [29]. During epidemics, due to the initial lack of protective immunity, *M. pneumoniae* infections can occur in humans of all age. Epidemics fade when the susceptible population gains protective immunity against the pathogen. However, younger individuals, who had not been exposed and thus did not gain protective immunity, were consequently infected during the post-epidemic time period. Further epidemiological investigations are warranted to yield more serological evidence for populations from different regions and periods and elucidate the biological and epidemiological reasons that contribute to this phenomenon.

Fulminant pneumonia accounted for 0.5–2% of all *M. pneumoniae* pneumonia cases and primarily affected young adults [30]. Two Chinese reports found that higher ICU admission rates (32.8%) and fatal *M. pneumoniae* pneumonia were be found in younger children [21, 31]. Moreover, in USA and Europe, the rates of ICU admission of hospitalized patients with *M. pneumoniae* pneumonia were reported as 10 and 16.3% [19, 32]. Here, during the whole period, only 14/1127 (1.2%) *M. pneumoniae* patients were treated in ICUs, with a median (IQR) age of 34 (37.75) years (range 15–83 years). Two factors may have contributed to the underreported number of ICU admissions: 1) nearly half of the detected patients were mildly symptomatic and treated as outpatients; 2) the severely ill children patients were immediately transferred to Children’s Hospital. A special phenomenon was observed that all the ICU patients positive with *M. pneumoniae* were admitted during the epidemic period (Table 3), along with the significantly higher incidence of *M. pneumoniae* RTIs in elderly and very elderly patients (Table 2). Although Khoury et al. retrospectively investigated *M. pneumoniae* patients from 2007 to 2012, covering the epidemic 2010–2012, the yearly distribution of the *M. pneumoniae* cases were not described [32]; thus these results do not enable a comparison of ICU admission rates during the epidemic. Nevertheless, both previous and our results indicated that *M. pneumoniae* should be considered as a possible pathogen in pneumonia patients admitted to the ICU in the setting of epidemic.

Limited by the retrospective design of the study, multivariable analysis, and time trend analysis could not be performed since more medical data (e.g., history of drug use, recent hospitalization, socio-economical factors and etc) could not be obtained for most of the patients. No information collected to analyze the effect of sources of the patients on the results is another limitation, since parts of the studied patients came from cities out of Beijing. Considering the most reliable diagnosis for *M. pneumoniae* RTIs would come from a combination of two or more separate laboratory methods, such as serology and PCR [33–36], another limitation of the study is the lack of serological results. Due to the much lower sensitivity, serological testing is actually not widely performed in our hospital, especially for adults [7]. Moreover, although all analyzed patients presented with RTIs, we still could not exclude the possible carriages in the upper respiratory tract [37]. However, the results of studies on carriages are inconsistent, from a low rate (< 3%) [38] to a relatively high proportion (13.5 and 4.6%) [39]. And the larger size and six-year consecutive detection strengthen our study.

## Conclusions

Different patterns of age distribution and seasonality of *M. pneumoniae* RTIs between epidemic (2011–2013) and post-epidemic (2014–2016) periods were firstly reported. Most of older patients and all of ICU admissions infected with *M. pneumoniae* occurred in epidemic period.

## Additional files

Additional file 1: **Figure S1.** Age distribution of total cases and *M. pneumoniae* infected patients. (DOCX 22 kb)

Additional file 2: **Figure S2.** Annual distribution of total cases and *M. pneumoniae* infected patients. (DOCX 24 kb)

Additional file 3: **Table S1.** Monthly analysis of *M. pneumoniae* infections in children and adults patients. Data were represented as n (%) in total cases, and as n (positivity, %) in *M. pneumoniae* cases. (DOCX 15 kb)

Additional file 4: **Figure S3.** Cases distribution and *M. pneumoniae* positivity in children and adults patients grouped by months. (DOCX 48 kb)

Additional file 5: **Table S2.** Analysis of *M. pneumoniae* infections in children and adults during epidemic and post-epidemic. Data were represented as n (%) in total cases, and as n (positivity, %) in *M. pneumoniae* cases. (DOCX 16 kb)

Additional file 6: **Figure S4.** Cases distribution and *M. pneumoniae* positivity in children and adults patients during epidemic and post-epidemic grouped by months. (DOCX 60 kb)﻿

Additional file 7: **Figure S5.** Cases distribution and *M. pneumoniae* positivity in epidemic and post-epidemic among age groups. (DOCX 62 kb)

## Abbreviations

Balf: bronchoalveolar lavage fluid
CAP: community-acquired pneumonia
EICU: Emergency ICU
FQ-PCR: the fluorescence quantitative PCR
hRNP: human ribonuclease protein
*M. pneumoniae*: *Mycoplasma pneumoniae*
RICU: Respiratory ICU
RTIs: respiratory tract infections
