# Supplementary figures and images for: Epidemiological characterization of respiratory tract infections caused by Mycoplasma pneumoniae during epidemic and post-epidemic periods in North China, from 2011 to 2016
Source: BMC Infect Dis. 2018 Jul 17;18:335. doi: 10.1186/s12879-018-3250-2 (PMC6050680; doi:10.1186/s12879-018-3250-2)

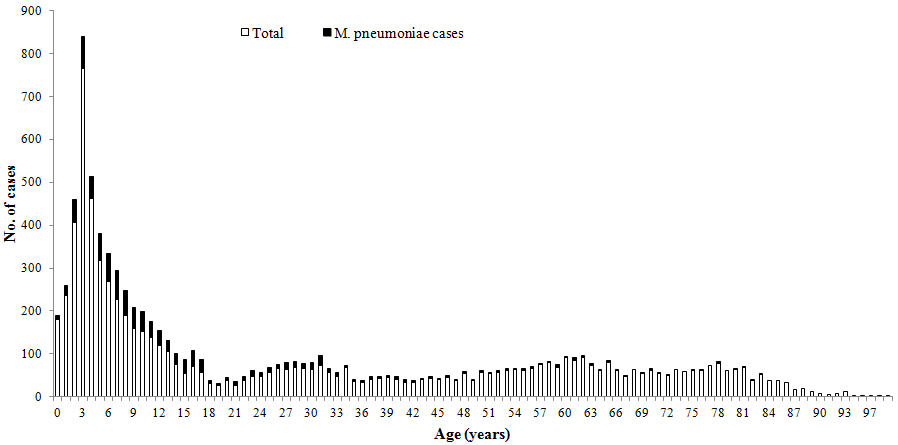

Supplement: Supplementary file 1 — Figure S1. Age distribution of total cases and M. pneumoniae infected patients. (DOCX 22 kb) [file 12879_2018_3250_MOESM1_ESM.docx]

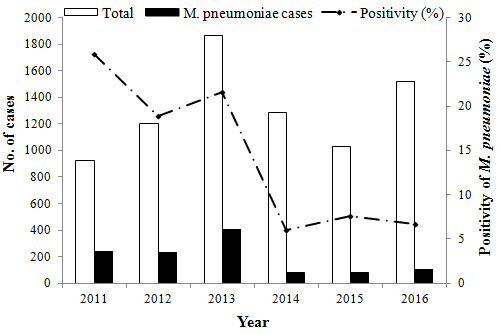

Supplement: Supplementary file 2 — Figure S2. Annual distribution of total cases and M. pneumoniae infected patients. (DOCX 24 kb) [file 12879_2018_3250_MOESM2_ESM.docx]

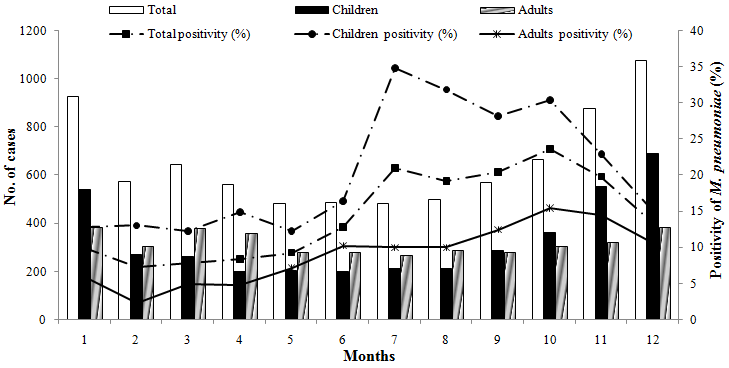

Supplement: Supplementary file 4 — Figure S3. Cases distribution and M. pneumoniae positivity in children and adults patients grouped by months. (DOCX 48 kb) [file 12879_2018_3250_MOESM4_ESM.docx]

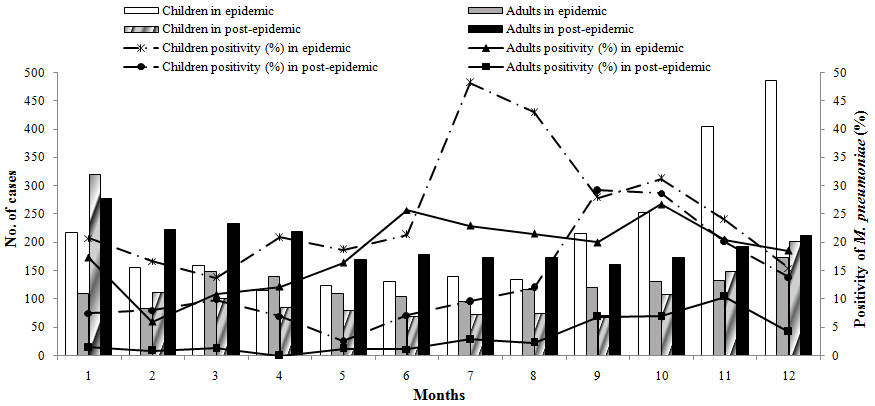

Supplement: Supplementary file 5 — Table S2. Analysis of M. pneumoniae infections in children and adults during epidemic and post-epidemic. Data were represented as n (%) in total cases, and as n (positivity, %) in M. pneumoniae cases. (DOCX 16 kb) [file 12879_2018_3250_MOESM5_ESM.docx]

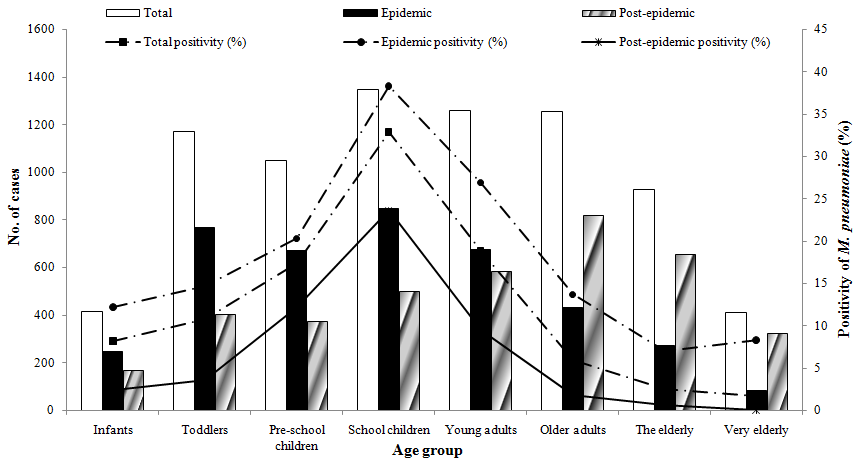

Supplement: Supplementary file 6 — Figure S4. Cases distribution and M. pneumoniae positivity in children and adults patients during epidemic and post-epidemic grouped by months. (DOCX 60 kb)﻿ [file 12879_2018_3250_MOESM6_ESM.docx]
