# Supplementary material for: Epidemiological characterization of respiratory tract infections caused by Mycoplasma pneumoniae during epidemic and post-epidemic periods in North China, from 2011 to 2016
Source: BMC Infect Dis. 2018 Jul 17;18:335. doi: 10.1186/s12879-018-3250-2 (PMC6050680; doi:10.1186/s12879-018-3250-2)
Supplement: Supplementary file 3 — Table S1. Monthly analysis of M. pneumoniae infections in children and adults patients. Data were represented as n (%) in total cases, and as n (positivity, %) in M. pneumoniae cases. (DOCX 15 kb) [file 12879_2018_3250_MOESM3_ESM.docx]

**Table S1**. Monthly analysis of *M. pneumoniae* infections in children and adults patients. Data were represented as n (%) in total cases, and as n (positivity, %) in *M. pneumoniae* cases.

| Month | Total | *M. pneumoniae* cases | Children | | Adults | | *P* value |
| --- | --- | --- | --- | --- | --- | --- | --- |
|  |  |  | Total | *M. pneumoniae* cases | Total | *M. pneumoniae* cases |  |
| 1 | 925 | 92 (9.9) | 538 | 69 (12.8) | 387 | 23 (5.9) | 0.001 |
| 2 | 574 | 42 (7.3) | 268 | 35 (13.1) | 306 | 7 (2.3) | < 0.001 |
| 3 | 642 | 51 (7.9) | 261 | 32 (12.3) | 381 | 19 (5.0) | 0.001 |
| 4 | 561 | 47 (8.4) | 201 | 30 (14.9) | 360 | 17 (4.7) | < 0.001 |
| 5 | 483 | 45 (9.3) | 203 | 25 (12.3) | 280 | 20 (7.1) | 0.058 |
| 6 | 484 | 62 (12.8) | 201 | 33 (16.4) | 283 | 29 (10.2) | 0.053 |
| 7 | 482 | 101 (21.0) | 212 | 74 (34.9) | 270 | 27 (10.0) | < 0.001 |
| 8 | 500 | 96 (19.2) | 210 | 67 (31.9) | 290 | 29 (10.0) | < 0.001 |
| 9 | 568 | 116 (20.4) | 287 | 81 (28.2) | 281 | 35 (12.5) | < 0.001 |
| 10 | 665 | 157 (23.6) | 361 | 110 (30.5) | 304 | 47 (15.5) | < 0.001 |
| 11 | 877 | 174 (19.8) | 553 | 127 (23.0) | 324 | 47 (14.5) | 0.003 |
| 12 | 1074 | 144 (13.4) | 688 | 103 (15.0) | 386 | 41 (10.6) | 0.05 |
| Total | 7835 | 1127 (14.4) | 3983 | 786 (19.7) | 3852 | 341 (8.9) | < 0.001 |

*P* value: statistical analyses were performed on *M. pneumoniae* cases between children and adults.
