# Supplementary material for: Epidemiological characterization of respiratory tract infections caused by Mycoplasma pneumoniae during epidemic and post-epidemic periods in North China, from 2011 to 2016
Source: BMC Infect Dis. 2018 Jul 17;18:335. doi: 10.1186/s12879-018-3250-2 (PMC6050680; doi:10.1186/s12879-018-3250-2)
Supplement: Supplementary file 7 — Figure S5. Cases distribution and M. pneumoniae positivity in epidemic and post-epidemic among age groups. (DOCX 62 kb) [file 12879_2018_3250_MOESM7_ESM.docx]

**Table S2**. Analysis of *M. pneumoniae* infections in children and adults during epidemic and post-epidemic. Data were represented as n (%) in total cases, and as n (positivity, %) in *M. pneumoniae* cases.

| Month | Epidemic | | | | *P* value | Post-epidemic | | | | *P* value |
| --- | --- | --- | --- | --- | --- | --- | --- | --- | --- | --- |
|  | Children | MP+ ^a^ children | Adults | MP+ adults |  | Children | MP+ children | Adults | MP+ adults |  |
| 1 | 217 | 45 (20.7) | 110 | 19 (17.3) | 0.555 | 321 | 24 (7.5) | 277 | 4 (1.4) | < 0.001 |
| 2 | 156 | 26 (16.7) | 83 | 5 (6.0) | 0.025 | 112 | 9 (8.0) | 223 | 2 (0.9) | 0.001 |
| 3 | 160 | 22 (13.8) | 148 | 16 (10.8) | 0.49 | 101 | 10 (9.9) | 233 | 3 (1.3) | 0.001 |
| 4 | 115 | 24 (20.9) | 140 | 17 (12.1) | 0.062 | 86 | 6 (7.0) | 220 | 0 (0) | < 0.001 |
| 5 | 123 | 23 (18.7) | 110 | 18 (16.4) | 0.731 | 80 | 2 (2.5) | 170 | 2 (1.2) | 0.595 |
| 6 | 131 | 28 (21.4) | 105 | 27 (25.7) | 0.443 | 70 | 5 (7.1) | 178 | 2 (1.1) | 0.02 |
| 7 | 139 | 67 (48.2) | 96 | 22 (22.9) | < 0.001 | 73 | 7 (9.6) | 174 | 5 (2.9) | 0.045 |
| 8 | 135 | 58 (43.0) | 116 | 25 (21.6) | < 0.001 | 75 | 9 (12.0) | 174 | 4 (2.3) | 0.003 |
| 9 | 215 | 60 (27.9) | 120 | 24 (20.0) | 0.117 | 72 | 21 (29.2) | 161 | 11 (6.8) | < 0.001 |
| 10 | 253 | 79 (31.2) | 131 | 35 (26.7) | 0.41 | 108 | 31 (28.7) | 173 | 12 (6.9) | < 0.001 |
| 11 | 404 | 97 (24.0) | 132 | 27 (20.5) | 0.476 | 149 | 30 (20.1) | 192 | 20 (10.4) | 0.014 |
| 12 | 486 | 75 (15.4) | 173 | 32 (18.5) | 0.34 | 202 | 28 (13.9) | 213 | 9 (4.2) | 0.001 |
| Total | 2534 | 604 (23.8) | 1464 | 267 (18.2) | < 0.001 | 1449 | 182 (12.6) | 2388 | 74 (3.1) | < 0.001 |

^a^ MP+: *M. pneumoniae* positive. *P* value: statistical analyses were performed on *M. pneumoniae* cases between children and adult
